# Crohn’s Disease-Associated and Cryptoglandular Fistulas: Differences and Similarities

**DOI:** 10.3390/jcm12020466

**Published:** 2023-01-06

**Authors:** Zhou Zhou, Laura F. Ouboter, Koen C. M. J. Peeters, Lukas J. A. C. Hawinkels, Fabian Holman, Maria F. Pascutti, Marieke C. Barnhoorn, Andrea E. van der Meulen-de Jong

**Affiliations:** 1 Department of Gastroenterology and Hepatology, Leiden University Medical Center, 2333 ZA Leiden, The Netherlands; 2 Department of Immunology, Leiden University Medical Center, 2333 ZA Leiden, The Netherlands; 3 Department of Surgery, Leiden University Medical Center, 2333 ZA Leiden, The Netherlands

**Keywords:** perianal fistulas, Crohn’s disease, cryptoglandular fistulas, differences and similarities, clinical practice

## Abstract

Perianal fistulas are defined as pathological connections between the anorectal canal and the perianal skin. Most perianal fistulas are cryptoglandular fistulas, which are thought to originate from infected anal glands. The remainder of the fistulas mainly arises as complications of Crohn’s disease (CD), trauma, or as a result of malignancies. Fistulas in CD are considered as a consequence of a chronic and transmural inflammatory process in the distal bowel and can, in some cases, even precede the diagnosis of CD. Although both cryptoglandular and CD-associated fistulas might look similar macroscopically, they differ considerably in their complexity, treatment options, and healing rate. Therefore, it is of crucial importance to differentiate between these two types of fistulas. In this review, the differences between CD-associated and cryptoglandular perianal fistulas in epidemiology, pathogenesis, and clinical management are discussed. Finally, a flow chart is provided for physicians to guide them when dealing with patients displaying their first episode of perianal fistulas.

## 1. Introduction

A perianal fistula is a pathological connection between the anorectal canal and the perianal skin [1]. Patients with perianal fistulas suffer a lot from anorectal pain, malodorous drainage, and sometimes fecal incontinence. These symptoms seriously affect the quality of life of these patients [2]. More than 90% of perianal fistulas are cryptoglandular fistulas which are thought to originate from infected anal glands. Acute infection of these glands results in the onset of perianal abscess and subsequent fistulas [3]. The second common type is Crohn’s disease (CD)-associated fistulas. CD, a chronic inflammatory disease, is characterized by segmental, transmural, and recurrent intestinal inflammation and fistulas are a very common and severe complication of CD [4]. In some cases, fistula development might coincide, or even precede the diagnosis of CD, and this rate is reported from 30.4% to 68.6% [5,6]. In these cases, identifying CD-associated fistulas from cryptoglandular fistulas might be challenging.

The ultimate goal for the treatment of perianal fistulas is to eradicate the fistula while preventing fecal incontinence [7]. Importantly, the management of cryptoglandular and CD-associated fistulas is different. CD-associated fistulas rely on the combination of pharmacological therapies and surgical interventions, while cryptoglandular fistulas are mainly treated with surgery [8,9]. Despite multiple treatment modalities, one-third of CD-associated fistulas remain unhealed and one-fifth of these patients finally require proctectomy [10]. Furthermore, high recurrence rates for both types of perianal fistulas, especially for CD-associated fistulas, increase the complexity of this disease [11,12].

The pathogenesis of fistula formation is not yet fully understood. The currently accepted view on cryptoglandular fistulas is that they result from inflammation of the anal glands, while CD-associated fistulas arise as a consequence of a dysregulated, chronic, and transmural inflammatory process in the distal bowel [13,14]. Further research is needed to refine the understanding.

Given that these two types of fistulas differ considerably in pathogenesis, complexity, treatment, and prognosis, it is crucial to differentiate between these two types of fistulas. When the luminal CD is not present, this will be challenging. In a recent systematic review, multiple diagnostic tests to distinguish CD-associated and cryptoglandular fistulas were compared, revealing that none of these tests alone show sufficient sensitivity [15]. In order to better understand the differences between these two types of fistulas, this review summarizes the current knowledge on their epidemiology, pathogenesis, and clinical management. Finally, a flow chart is provided to give guidance to physicians when dealing with patients displaying their first episode of perianal fistulas.

## 2. Classification of Perianal Fistulas

**Figure 1:**
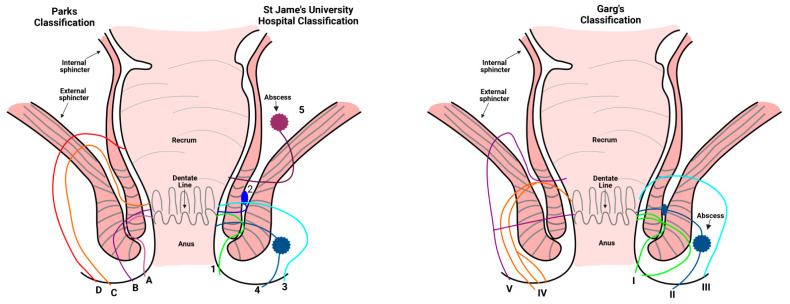
The classification of perianal fistulas. Parks classification: A. intersphincteric fistulas; B. transsphincteric fistulas; C. suprasphincteric fistulas; D. extrasphincteric fistulas. St James’s University Hospital classification: Grade 1. simple linear intersphincteric fistulas; Grade 2. intersphincteric fistulas with abscesses; Grade 3. transsphincteric fistulas; Grade 4. transsphincteric fistulas with abscesses; Grade 5. supralevator and translevator fistulas. Garg’s classification: I. low liner intersphincteric and transsphincteric fistulas; II. low intersphincteric and transsphincteric fistulas; III. high liner transsphincteric fistulas; IV. complex high transsphincteric fistulas; V. suprasphincteric infralevator fistulas.

The commonly accepted classification of perianal fistulas is based on the surgical anatomy described by Parks and colleagues [16]. In the Parks classification (Figure 1), the external sphincter is used as the keystone to classify perianal fistulas into four groups: intersphincteric, transsphincteric, suprasphincteric and extrasphincteric fistulas. Perianal fistulas can be further classified into simple and complex fistulas. Simple fistulas are described as being low transsphincteric or intersphincteric, involving less than 30% of the external anal sphincter, while the complex fistulas include high transsphincteric, suprasphincteric, extrasphincteric fistulas, and horseshoe fistulas [17]. Magnetic resonance imaging (MRI) is considered as the optimal technique for distinguishing complex from simple perianal fistulas [18]. MRI exactly displays the relationship between fistulas and the pelvic structure and enables the identification of abscesses [1]. Based on MRI imaging, St James’s University Hospital classifies fistulas into five grades (Figure 1) [19]. Lastly, the Garg classification combines Parks and St James’s University Hospital classifications. It divides fistulas into five grades based and validated on the clinical experience, MRI, operative findings, and follow-up of 440 patients (Figure 1). Grade I-II are simple fistulas which can be treated by fistulotomy, while grade III-V are complex fistulas, in which sphincter saving management should be applied and fistulotomy should be avoided because of the risk of incontinence [20,21]. Garg’s classification is therefore useful for both radiologists and surgeons.

## 3. Epidemiology

The reported incidence of perianal fistulas varies substantially. Zanotti and colleagues searched the European hospital databases in four different countries and concluded that the incidence of perianal fistulas ranges from 1.2 to 2.8/10,000/year in Europe. However, they did not differentiate between cryptoglandular and CD-associated perianal fistulas [22]. A systematic literature review indicated that the most predominant perianal fistulas are cryptoglandular and CD-associated fistulas, whose estimated incidence in Europe is 0.86 and 0.21/10,000/year, respectively [23]. In the United States, the annual incidence was 20,000 to 25,000 cases [24]. In China, perianal fistulas make up 1.67 to 3.60% of anorectal diseases [25]. However, due to the limited reports in different areas, it is difficult to ascertain that the incidence of perianal fistulas differs from geographical region. Additional data from a study in England reported that the overall rate of fistula formation following anorectal abscess was 17.2% (27,349/158,713) within a 15-year (from 1997 to 2012) follow-up period [26]. Sub-classification on the cause of fistula formation following an anal abscess revealed that the rate of cryptoglandular fistulas was 15.5% and the rate of CD-associated fistulas was 47.2%. This suggests that CD is a risk factor for the development of fistulas in patients with an abscess.

For CD, it is reported that 13.9% to 28.8% of patients suffer from the perianal fistulizing disease [27,28]. Moreover, in patients with inflammation of the rectum, this number increases to 92% [29]. In the Netherlands, the data of the inflammatory bowel disease (IBD) South-Limburg (IBDSL) cohort showed that 13.9% (161/1162) of the CD patients developed a perianal fistula during the course of their disease, while the cumulative 5-year perianal fistula rate varied between 10.3% and 14.1% (from 1991 to 2011). The overall cumulative probability of developing a perianal fistula increases from 8.3% after 1 year to 11.6% after 5 years and 15.8% after 10 years [5], indicating that the risk of developing fistulas increases with a prolonged disease duration. In the Hong Kong territory-wide IBD registry, it was found that among 981 CD patients, 283 (28.8%) had perianal involvement, including perianal abscesses, perianal fistulas, fissures, and anal strictures, of which perianal fistulas were by far the most commonly observed (84.8%, 240/283) [30]. Notably, the introduction of biologics (anti-tumor necrosis factor (TNF)-α therapy) led to a revolution in the treatment of fistulizing CD since 1998. The medical records of residents in Olmsted Country from 1970 to 2010 showed that the cumulative incidence of perianal or rectovaginal fistulas was lower in CD patients diagnosed in the biologic era (1998 or later) compared with the pre-biologic era (before 1998) (25.8% vs. 14.5%) [31]. Conversely, data from the IBDSL cohort indicated no significant difference in the cumulative 5-year perianal fistula rate between eras (14.1% in the 1991–1998 era, 10.4% in the 1999–2005 era, 10.4% in the 1999–2005 era) [5]. However, it was obvious that the incidence was higher before 1998 compared to the biologic era.

It is also interesting that gender is a risk factor for the incidence of perianal fistulas. An evaluation of the Swiss IBD Cohort Study, including 1600 CD patients, showed that females had a lower risk of developing perianal fistulas (univariate analysis, RR = 0.727, *p* = 0.002) [32]. In agreement with this data, data from the Hong Kong territory-wide IBD registry and Korea cohort also indicated that the majority of CD patients with perianal diseases were males (78.8%, 223/283 and 73.8%, 343/465, respectively) [30,33]. In accordance with the CD-associated fistulas, cryptoglandular fistulas also occur 1.8 to 9 times more often in male patients [34,35]. In infants, this ratio is even higher, with 97.5% (478/490) of the anal fistulas being present in male patients [36]. This difference may be explained by excess androgen and higher sphincter tone in males [37].

To summarize, the incidence of cryptoglandular fistulas varies between countries. For CD-associated fistulas, the incidence increases with prolonged disease duration and may decrease after the application of biologics. For both types of fistulas, presence is more dominant in male patients.

## 4. Pathogenesis

The pathogenesis of perianal fistulas is not fully understood. Although there is some overlap in the etiology of cryptoglandular fistulas and CD-associated fistulas, there seem to be considerable differences. In general, there are four mechanisms that may play an important role in the development of fistulas: anal gland inflammation, intestinal inflammation, epithelial-mesenchymal transition (EMT), and disturbed microbiota.

### 4.1. Anal Gland Inflammation

For cryptoglandular fistulas, the theory of Parks is widely accepted which states that perianal fistulas result from inflammation of the anal glands [13]. Anal glands are found at varying depths in the anal canal wall between the layers of the internal and external sphincter, called the intersphincteric plane. On average, there are six glands (ranging from three to ten) which are fairly evenly distributed in the anal circumference at the dentate line [38].

Infected glands are caused by obstruction of the bacteria, fecal material, or foreign matter which results in stasis, bacterial overgrowth, and the subsequent formation of abscesses. The original report from Parks in 1961 showed that eight out of 30 perianal fistulas cases displayed gross cystic dilatation of an anal gland. Furthermore, 13 cases showed anal-gland epithelium lining the intersphincteric abscess or part of the fistula tracts, and seven cases had an anal gland present which was not part of the fistula tract. Therefore, the authors concluded that 90% of the perianal fistulas started from an infection of the anal gland [13]. However, this view changed in subsequently published studies. In 1967, it was reported that intersphincteric abscesses were only found in eight of the 28 abscess cases, indicating that Parks theory does not apply to all cases [39]. This was further strengthened by another study that found that there was no anal gland tissue with mucin-producing cells in any of the fistulas among 53 specimens from 44 cryptoglandular fistulas patients [40]. Overall, this indicates that anal gland infection plays a role in only some cases, or certain phases of the formation of cryptoglandular fistulas.

Regarding CD-associated fistulas, hypotheses have been postulated that fistulas originate from anal gland abscesses as well [11,41,42], but none of these studies provided solid evidence. On the contrary, it was reported that fistulas in six out of nine reported CD patients were associated with infiltration of the internal sphincter without evidence of diseased anal glands [43]. Moreover, CD patients can suffer from cryptoglandular abscesses/fistulas, but these fistulas are superficial and are not related to active anorectal CD [44].

In summary, anal gland inflammation may contribute to the onset of cryptoglandular fistulas, which is considered as the continuous phase of anorectal abscesses. On the other hand, it seems to play a limited role in the development of CD-associated fistulas.

### 4.2. Intestinal Inflammation

Intestinal inflammation in CD results from dysregulated mucosal immune responses to microbiota in the intestinal mucosa [45]. It is characterized by an altered response of innate and adaptive immune cells, as well as increased production of inflammatory cytokines. This process is key in the pathogenesis underlying CD, but it may also play a role in the formation and perpetuation of fistulas [46]. Several immunological factors involved in perianal fistulizing disease are described, but research in this field remains scarce. In CD-associated fistulas, activated CD4^+^ T cells appear to be crucial. Data from our group published by Bruckner and colleagues showed an increased CD4^+^:CD8^+^ T cell expression ratio in peripheral blood mononuclear cells (PBMC) from CD fistula patients compared to CD patients without a fistula and compared to healthy controls [47]. Earlier, Maggi and colleagues also described this increased ratio in fistula curettage and PBMCs from CD fistula patients. Additionally, CD4^+^CD161^+^ T cells were accumulated in CD fistulas compared to peripheral blood, which was reduced upon local anti-TNF-α therapy [48]. Contrastingly, van Unen and colleagues described fewer CD4^+^ T cells in the fistula tract compared to the rectum, highlighting a role for myeloid cells [49]. At the European Crohn’s and Colitis Organization (ECCO) in 2021, data presented of in-depth immunophenotyping using ‘Cytometry by time-of-flight’ (CyTOF) compared CD-associated fistulas with cryptoglandular fistulas. Although CD66a^+^ granulocytes were highly abundant in both types of fistulas, more cells from lymphoid origin were identified in CD-associated fistulas, while in cryptoglandular tracts myeloid cells were enriched [50]. It is important to consider that almost all CD patients participating in these studies were treated with immunomodulators and/or anti-TNF-α therapy that might have changed the immune composition in the CD-associated fistulas. Nevertheless, it remains elusive how the phenotypically complex immune cells are (co)-localized and interact around the fistula tract. New advances, such as ‘Imaging Mass Cytometry (IMC)’ now offer the opportunity to visualize multiple lineages simultaneously and explore this further.

The level of cytokines, mostly produced by immune cells, but also by for example stromal cells, is indicative of the inflammatory fistula environment. In cryptoglandular fistulas, IL-1β is one of the most abundant cytokines. Onkelen and colleagues found that 93% (25/27) of the cryptoglandular fistulas expressed IL-1β. They also found that IL-8, IL-12p40, and TNF-α were detected in 70%, 33%, and 30% of the perianal fistula samples, respectively [51]. IL-1β was shown to be more expressed in the distal than the proximal part of the fistulas (2.01 vs. 1.33), while the IL-8 had a reverse expression pattern (3.60 vs. 4.34). Still, both were overexpressed compared with the normal anal mucosa. This suggests that the inflammatory pattern might be different in the proximal than in the distal part of the fistula tracts [52]. Our unpublished data showed that the expression of interleukin (IL)-12, interferon-gamma (IFN-γ), IL-13, IL-17, IL-1β, IL-4, IL-5, IL-6, IL-8, and TNF-α were significantly elevated in the CD-associated fistula tract compared to the non-inflamed rectum wall from the same patients. In contrast, oncostatin M levels were found to be higher in the non-inflamed rectum compared to the perianal fistula [53]. Unfortunately, data comparing the cytokine levels in CD and cryptoglandular fistulas are still missing. Further studies are necessary to draw a final conclusion on the cytokine patterns in the two types of perianal fistulas.

### 4.3. Epithelial-Mesenchymal Transition

Next to inflammation, epithelial-mesenchymal transition (EMT) probably contributes to the formation of perianal fistulas. EMT refers to the transformation of epithelial to mesenchymal cells, in which cells lose their epithelial features, such as cell polarity and cell-cell adhesions, and gain mesenchymal characteristics, including migratory and invasive properties. EMT is both involved in physiological processes, such as embryogenesis and tissue repair, but also in diseases, including cancer and fibrosis [54]. Previous work has revealed that CD-associated fistulas might originate from an epithelial defect that occurs during chronic inflammation, followed by EMT as discussed below [14].

Firstly, decreased expression of E-cadherin accompanied by increased levels of transforming growth factor beta (TGF-β) and β6-integrin were observed in the intestinal epithelial cells (IECs) covering the CD-associated fistulas tracts, when compared to normal IECs [55], suggesting mesenchymal changes in these cells. This kind of IECs, called “transition cells” (TCs), still express epithelial markers cytokeratin 8 (CK8) and cytokeratin (CK20), indicating their epithelial origin. However, they also expressed mesenchymal markers and lacked tight junctions, which was followed by an increased migratory capacity [55]. TGF-β is a main inducer of EMT and regulates many EMT-related cytokines and transcription factors. For example, SNAIL and SLUG, two transcription factors which are able to repress the expression of the epithelial adhesion protein E-cadherin, were highly expressed in CD-associated fistulas [56]. Furthermore, TGF-β-induced IL-13 expression promoted the expression of genes associated with cell invasion in CD-associated fistulas [57]. TNF-α, which is in high concentration present in both the inflamed rectum and fistula samples, can induce EMT in IECs but is also capable of inducing the expression of the main EMT regulator TGF-β [58]. Our work suggests that CD3^+^ T cells are the major sources of TNF-α in CD-associated fistulas, thereby driving EMT [47]. Additionally, elevated expression of matrix metalloproteinases (MMPs), especially MMP-3 and MMP-9, was documented in CD-associated fistula tracts. These MMPs are able to enhance extracellular matrix (ECM) degradation, release growth factors, induce EMT and finally stimulate fistula formation [59].

It seems that EMT does not play a (major) role in the pathological process of cryptoglandular fistulas. However, in a prospective pilot study, increased expression of TGF-β, Vimentin, Zeb-1, and SNAIL were detected, accompanied by decreased levels of E-cadherin in perianal fistulas compared to the normal adjacent anal mucosa. The sample size of this study was limited and therefore more and larger-scale studies are needed to confirm these findings [52].

### 4.4. Microbiota

Although more knowledge became available in the last decade about the role of the microbiome in the pathogenesis of IBD [60], the function of the microbiome in the etiology of perianal fistulas remains controversial. In CD-associated fistulas, Haac and colleagues reported that higher numbers of Achromobacter and Corynebacterium, as well as lower levels of Bifidobacterium, were found in fistula tracts compared to fecal samples by using 16S rRNA [61]. Our unpublished data, using 16S rRNA sequencing, also showed a fistula-specific bacterial composition when compared to feces. However, Tozer and colleagues elucidated that bacteria were rarely found in the tract of a chronic perianal fistula, including 20 CD-associated and 18 cryptoglandular perianal fistulas, by using fluorescent in situ hybridization (FISH), gram staining, and scanning electron microscopy [62]. On the other hand, the association of a c-insertion mutation of the NOD2 gene with fistulizing CD emphasizes the importance of pathways triggered by bacterial components in the etiology of perianal fistulas [63]. Furthermore, muramyl dipeptide (MDP), the minimal essential structure of bacterial peptidoglycan, which forms the cell wall of most bacteria, could stimulate the expression of EMT-associated genes in IECs and CD-associated fistula colonic lamina propria fibroblasts (CLPFs). This implies that bacteria could start EMT to provoke an immune response, and thereby play a role in the pathogenesis of CD-associated fistulas via several pathways [58].

As for cryptoglandular fistulas, a recent study revealed that anal glands in fistulas lack goblet cells, which can result in the reduction of mucins secretion and, therefore, the columnar epithelial cells will be directly exposed to (pathogenic) microorganisms [64]. Previous work showed indeed that “gut-specific Bacteroides” in the pus of the abscess was more frequent in patients with fistulas compared to those without [65,66]. In contrast, several other retrospective studies reported that the presence of a positive culture of pus was not associated with fistula formation [67,68]. Moreover, bacterial remnants have been described to contribute to the ongoing inflammation in fistulas. Peptidoglycan was detected in 90% (9/10) of the cryptoglandular fistulas, whereas a host response to peptidoglycan was detected in 60% of these patients. Nevertheless, they could only identify bacterial RNA in one fistula using 16S rRNA sequencing [69]. This suggests that bacterial remnants (peptidoglycan) have a role in chronic inflammation even in the absence of local bacterial replication or more sensitive techniques are needed to identify bacteria.

**Figure 2:**
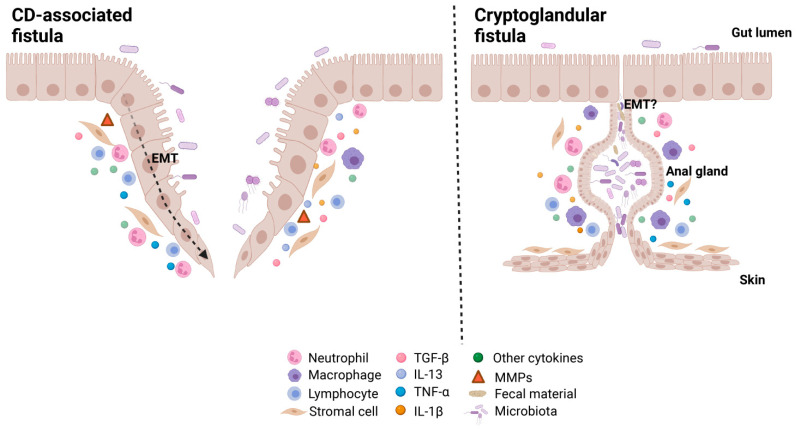
Proposed mechanism of the pathogenesis of CD-associated perianal fistulas and cryptoglandular perianal fistulas. EMT: epithelial-mesenchymal transition; TGF-β: transforming growth factor beta; IL-13: interleukin 13; TNF: tumor necrosis factor; IL-1β: interleukin 1 beta; MMPs: matrix metalloproteinases.

In short, multiple factors seem to be involved in the pathogenesis of perianal fistulas (Figure 2). It seems that acute infection starts in perianal glands in patients with cryptoglandular fistulas, followed by the subsequent formation of an abscess. This might lead to a draining tract that eventually forms a fistula, while pro-inflammatory cytokines contribute to continued tissue damage. Microbiota and its remnants (peptidoglycan, for example) trigger and maintain inflammation. In CD-associated fistulas, it seems that chronic intestinal inflammation, resulting in abundant secretion of cytokines like TNF-α and TGF-β, together with, epithelial defects contribute to EMT [47]. Microbiota and remnants are thought to participate in both inflammation and EMT. Activated MMPs lead to further tissue damage, inflammation, and fistula formation.

## 5. Treatment

The treatment of perianal fistulas, especially complex fistulas, is still a major challenge for both surgeons and gastroenterologists. For cryptoglandular fistulas, no specific medication is registered, apart from antibiotics for treating infectious complications from the fistulas. However, the overall management of CD-associated fistulas requires close collaboration between gastroenterologists and surgeons. The pharmacological therapy of CD-associated perianal fistulas is an important backbone, with the main goal to control local (fistula) and luminal inflammation. Treatment should be tailored to the individual characteristics of each patient [70]. Anti-TNF remains the first-line intervention for fistulizing CD. With high serum anti-TNF-α levels (≥10 mcg/mL), patients achieve improved outcomes [71]. Unfortunately, about one-third of patients do not respond to anti-TNF therapy at all, and half will lose response within one year [72]. Only 25% of patients with perianal fistulizing CD experience sustained fistula closure with biologic therapy [73]. Other biologics, like anti-p40 (IL-12/IL-23) and integrin receptor antagonists, might be beneficial for the treatment of CD-associated fistula patients as well [74,75], but more data are required.

Surgical therapy is the treatment of choice for cryptoglandular fistulas and is also crucial for CD-associated fistulas. The goal of the surgical intervention is to eradicate the fistula while preventing fecal incontinence. Fistulotomy is recommended for symptomatic simple cryptoglandular and superficial or low fistulas (less than 1/3 of external sphincter involvement) without incontinence or proctitis in patients with CD (Garg Grad I–II) [76]. Healing rates after fistulotomy range from 74% to 100% in these patients [77]. Complex perianal fistulas (in both non-CD and CD patients) (Garg Grade III–V) require different surgical approaches. A loose seton is an effective treatment to facilitate drainage of the abscess. For patients with multiple fistula tracts and severe recurrent fistulas or abscesses within 2 years, a diverting stoma is considered. Both seton and stoma can prevent the recurrence of abscess formation, reduce perianal disease activity and increase the success rate for the following surgery [78]. However, they cannot heal the fistulas. Advancement flap repair is a technique that is often used for fistula closure of cryptoglandular fistulas. A systematic review including 30 studies comprising 1295 patients reported that the overall success of this advancement flap procedure was 74.6% [79]. Ligation of the intersphincteric fistula tract (LIFT) is another commonly used procedure for complex transsphincteric fistulas. This procedure closes the internal and external opening securely and removes infected cryptoglandular tissue via an intersphincteric approach [80]. The reported healing rate varies between 40% and 95% [81]. Other techniques, such as fibrin glue, anal fistula plug (AFP), video-assisted anal fistula treatment (VAAFT), and fistula laser closure (FiLaC), have also been developed, but more evidence is needed to evaluate their practicality and superiority [79,82,83,84]. It was reported that the majority of CD patients with perianal fistulas underwent at least once procedure [85]. Interestingly, the rate of proctectomy, which is considered as the last option for refractory and uncontrolled perianal fistulas, decreased in the biologic era compared with the pre-biologic era [31,86]. This indicates that biologics might have a protective effect on the natural history of perianal fistulas. Additionally, Lansdorp and colleagues found that hyperbaric oxygen therapy (HBOT) was beneficial for refractory CD-associated fistulas. Compared to the baseline, they achieved clinical and radiological improvement after one-year follow-up [87]. This might open new opportunities for the treatment of CD-associated fistulas. Thus, multimodal treatment for CD patients with perianal fistulas is very important.

Recently, a multicenter, patient preference study conducted in nine hospitals in The Netherlands and one hospital in Italy declaimed that short-term (4-month) anti-TNF treatment combined with surgical closure achieved a higher MRI healing rate compared with one-year anti-TNF treatment (32% vs. 9%, *p* = 0.005) in patients with CD-associated fistulas. However, clinical closure was not significantly different in these two groups (68% vs. 52%, *p* = 0.076) [88]. Furthermore, mesenchymal stromal cells (MSCs) have been shown to have therapeutic effects on patients with CD-associated perianal disease [89]. Alofisel/Cx601 is the first allogeneic stem cell therapy for the perianal fistulizing CD which was approved by the European Medicines Agency (EMA) for the treatment of refractory perianal fistulas in 2018 [90]. It consists of allogeneic (donor-derived) expanded adipose-derived stem cells (eASCs), which have immunomodulatory and anti-inflammatory effects at inflammation sites. In a multicenter trial, 212 CD patients with refractory, draining, and complex perianal fistulas were randomized to Alofisel and control group. After 52 weeks of treatment, Alofisel group achieved higher combined remission (clinical assessment and confirmed by MRI) (56.3%) compared to the placebo group (38.6%) [91]. Currently, in The Netherlands, we only prescribe Alofisel if surgical closure failed before because of the high costs and the relatively low success rate in comparison to the placebo. If repeating MSC injections, other types of MSCs (allogeneic vs. autologous, bone marrow versus adipose-derived) [92,93] might improve the outcomes. This could be the new subject for future studies.

In summary, the therapies differ for CD-associated and cryptoglandular fistulas. Therefore, it is of clinical significance to diagnose CD-associated fistulas from cryptoglandular fistulas as early as possible. Patients without any luminal inflammation, but with complex, recurrent fistulas are recommended to be treated as CD-associated perianal fistulas in our opinion, starting with anti-TNF-α therapy in combination with surgical therapy.

## 6. Distinction between Cryptoglandular and CD-Associated Fistulas: Clinical Practice

**Figure 3:**
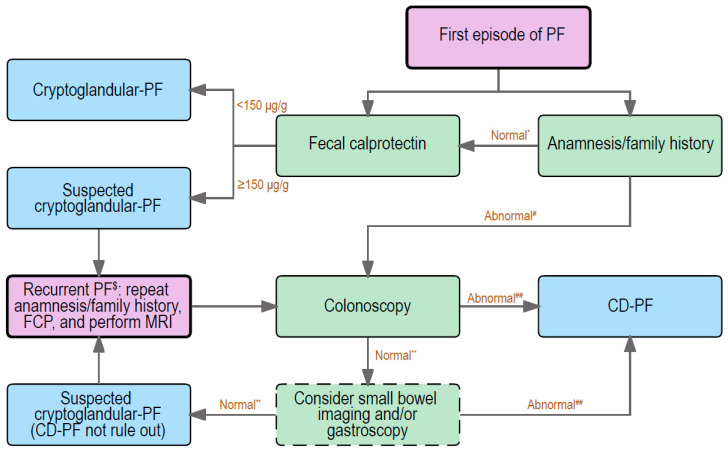
Flow chart to deal with a patient displaying his/her first episode of perianal fistulas. PF: perianal fistula; CD-PF: CD-associated perianal fistula. *: No CD-associated symptoms, no IMDs, and no IBD relatives. ^#^: CD-associated symptoms, or/and IMDs, or/and IBD relatives. **: no intestinal inflammation. ^##^: intestinal inflammation. ^$^: within one year. FCP: fecal calprotectin; MRI: magnetic resonance imaging.

Distinguishing cryptoglandular from CD-associated fistulas is usually not very difficult, since luminal CD-associated symptoms are typically present. However, in some patients with complex perianal disease, without symptoms associated with luminal CD, this can be challenging. Because early diagnosis of CD gives the opportunity to apply an early intensive treatment that might improve the outcome and reduce future surgical costs [94], it is recommended to confirm or exclude the presence of CD as soon as possible. Figure 3 shows our suggested diagnostic algorithm in patients with the first episode of a perianal fistula.

It is recommended to start with an extensive anamnesis, focused on luminal CD, extra-intestinal manifestations of CD, the presence of other co-morbidities, e.g., immune-mediated diseases (IMDs), and the patients’ family history. The anamnesis should be repeated at every occurrence of a new perianal abscess/fistula. The common symptoms of CD include (bloody) diarrhea, abdominal cramps and tenderness, fatigue, loss of appetite, and weight loss. These symptoms should be absent in patients with cryptoglandular fistulas. Moreover, it is reported that IMDs, such as rheumatologic disorders, intrinsic asthma, psoriasis, and spondylarthritis, are more frequently observed in IBD patients [95]. Therefore, patients with IMDs have a higher chance to be suspected as IBD-related, since IBD and IMDs share common susceptibility genes [96]. Family history is a clear high-risk factor and a strong predictor for the development of IBD. The risk of CD in first-degree relatives of a CD patient is almost eight-fold higher compared with families without IBD [97,98]. Physical examination is recommended to assess the presence and the number of external openings and active drainage, although this can be difficult due to the pain and sphincter spasm [99]. Imaging may contribute to the exact location and extent of the fistula(s), and to classify the perianal fistulas. If imaging is desired, MRI is the preferred choice, especially for complex and recurrent fistulas, because it has the highest accuracy compared to clinical examination and anal endosonography [1,100]. If medical history reveals no IBD-associated symptoms, no other immune diseases, and a negative family history, together with a first episode of a simple fistula, a cryptoglandular fistula would be most likely (Figure 3).

Fecal calprotectin (FCP) is significantly increased in patients with CD-associated fistulas, with and without mucosal inflammation, when compared with cryptoglandular fistulas (FCP cut-off value ≥ 150 µg/g, AUC = 0.9000, 81% sensitivity and 89% specificity) [101]. Even in fistulizing CD patients without mucosal inflammation, FCP was still higher compared to patients with cryptoglandular fistulas (FCP cut-off value ≥ 150 µg/g, AUC = 0.857, 67% sensitivity and 90% specificity) [101]. Although above results are clear, FCP is not a 100% discriminator between non-CD and CD fistulas. Therefore, we recommend to perform a FCP in all patients with a presentation of a first episode of a perianal fistula (Figure 3), because FCP is also an important follow-up marker if the patient is diagnosed with CD in (the near) future. Meanwhile, C reactive protein (CRP) is also a commonly used marker for inflammation in serum, but CRP levels cannot discriminate between CD- and non-CD-associated fistulas in case of ongoing active inflammation [102].

If the perianal fistula does not heal after a surgical intervention, the physician should repeat the anamneses and FCP after 3 months. For patients with an anamnesis suggestive of CD, and/or IMDs involvement, and/or a family history of IBD, and for patients who have a recurrent fistula, a colonoscopy is recommended. These patients should be additionally discussed in a multidisciplinary setting with at least a surgeon, gastroenterologist, and radiologist present. If there are no signs of inflammation in the colon and terminal ileum, and the suspicion for CD is still high, the team can consider performing small bowel imaging to detect intestinal inflammation (Figure 3). In this case, the patient should be monitored closely as long as the perianal fistula is active.

## 7. Conclusions

In conclusion, perianal fistulas remain a major clinical challenge due to a poor understanding of the pathogenesis, limited treatment options, and high recurrence rates. Although they share some characteristics, cryptoglandular and CD-associated perianal fistulas are two different entities, and each should be approached in a different way. For clinicians, it is important to make an accurate distinction, to facilitate personalized therapies and multidisciplinary collaboration. Patients without any luminal inflammation, but with complex, recurrent perianal fistulas, are recommended to be treated as CD-associated perianal fistulas and to start with anti-TNF-α therapy with good trough levels. In the future, deeper exploration of the pathogenesis of both types of perianal fistulas will help the development of new therapeutic targets. Multimodal treatments and combining different pharmacological therapies might also achieve better outcomes.

## Data Availability

Data supporting the results of the study will be available on request.

## References

[1] de Miguel Criado J., del Salto L.G., Rivas P.F., del Hoyo L.F., Velasco L.G., de las Vacas M.I., Marco Sanz A.G., Paradela M.M., Moreno E.F. (2012). MR imaging evaluation of perianal fistulas: Spectrum of imaging features. Radiogr. A Rev. Publ. Radiol. Soc. N. Am. Inc.

[2] Vogel J.D., Johnson E.K., Morris A.M., Paquette I.M., Saclarides T.J., Feingold D.L., Steele S.R. (2016). Clinical Practice Guideline for the Management of Anorectal Abscess, Fistula-in-Ano, and Rectovaginal Fistula. Dis. Colon Rectum.

[3] Whiteford M.H. (2007). Perianal abscess/fistula disease. Clin. Colon Rectal Surg..

[4] Hendrickson B.A., Gokhale R., Cho J.H. (2002). Clinical aspects and pathophysiology of inflammatory bowel disease. Clin. Microbiol. Rev..

[5] Gottgens K.W., Jeuring S.F., Sturkenboom R., Romberg-Camps M.J., Oostenbrug L.E., Jonkers D.M., Stassen L.P., Masclee A.A., Pierik M.J., Breukink S.O. (2017). Time trends in the epidemiology and outcome of perianal fistulizing Crohn’s disease in a population-based cohort. Eur. J. Gastroenterol. Hepatol..

[6] Mizushima T., Ota M., Fujitani Y., Kanauchi Y., Iwakiri R. (2021). Diagnostic Features of Perianal Fistula in Patients With Crohn’s Disease: Analysis of a Japanese Claims Database. Crohn’s Colitis 360.

[7] Sica G.S., Di Carlo S., Tema G., Montagnese F., Del Vecchio Blanco G., Fiaschetti V., Maggi G., Biancone L. (2014). Treatment of peri-anal fistula in Crohn’s disease. World J. Gastroenterol..

[8] Limura E., Giordano P. (2015). Modern management of anal fistula. World J. Gastroenterol..

[9] Kotze P.G., Shen B., Lightner A., Yamamoto T., Spinelli A., Ghosh S., Panaccione R. (2018). Modern management of perianal fistulas in Crohn’s disease: Future directions. Gut.

[10] Bell S.J., Williams A.B., Wiesel P., Wilkinson K., Cohen R.C., Kamm M.A. (2003). The clinical course of fistulating Crohn’s disease. Aliment Pharm..

[11] Marzo M., Felice C., Pugliese D., Andrisani G., Mocci G., Armuzzi A., Guidi L. (2015). Management of perianal fistulas in Crohn’s disease: An up-to-date review. World J. Gastroenterol..

[12] Mei Z., Wang Q., Zhang Y., Liu P., Ge M., Du P., Yang W., He Y. (2019). Risk Factors for Recurrence after anal fistula surgery: A meta-analysis. Int. J. Surg..

[13] Parks A.G. (1961). Pathogenesis and treatment of fistuila-in-ano. Br. Med. J..

[14] Scharl M., Rogler G. (2014). Pathophysiology of fistula formation in Crohn’s disease. World J. Gastrointest. Pathophysiol..

[15] Chin Koon Siw K., Engel J., Visva S., Mallick R., Hart A., de Buck van Overstraeten A., McCurdy J.D. (2022). Strategies to Distinguish Perianal Fistulas Related to Crohn’s Disease From Cryptoglandular Disease: Systematic Review With Meta-Analysis. Inflamm. Bowel Dis..

[16] Parks A.G., Gordon P.H., Hardcastle J.D. (1976). A classification of fistula-in-ano. Br. J. Surg..

[17] Steele S.R., Kumar R., Feingold D.L., Rafferty J.L., Buie W.D. (2011). Practice parameters for the management of perianal abscess and fistula-in-ano. Dis. Colon Rectum.

[18] Sahni V.A., Ahmad R., Burling D. (2008). Which method is best for imaging of perianal fistula?. Abdom Imaging.

[19] Morris J., Spencer J.A., Ambrose N.S. (2000). MR imaging classification of perianal fistulas and its implications for patient management. Radiographics.

[20] Garg P. (2018). Garg Classification for Anal Fistulas: Is It Better than Existing Classifications?—A Review. Indian J. Surg..

[21] Garg P. (2017). Comparing existing classifications of fistula-in-ano in 440 operated patients: Is it time for a new classification? A Retrospective Cohort Study. Int. J. Surg..

[22] Zanotti C., Martinez-Puente C., Pascual I., Pascual M., Herreros D., Garcia-Olmo D. (2007). An assessment of the incidence of fistula-in-ano in four countries of the European Union. Int. J. Color. Dis..

[23] Garcia-Olmo D., Van Assche G., Tagarro I., Diez M.C., Richard M.P., Khalid J.M., van Dijk M., Bennett D., Hokkanen S.R.K., Panes J. (2019). Prevalence of Anal Fistulas in Europe: Systematic Literature Reviews and Population-Based Database Analysis. Adv.

[24] Nelson R. (2002). Anorectal abscess fistula: What do we know?. Surg. Clin North Am.

[25] Li J., Yang W., Huang Z., Mei Z., Yang D., Wu H., Wang Q. (2016). Clinical characteristics and risk factors for recurrence of anal fistula patients. Zhonghua Wei Chang Wai Ke Za Zhi.

[26] Sahnan K., Askari A., Adegbola S.O., Tozer P.J., Phillips R.K.S., Hart A., Faiz O.D. (2017). Natural history of anorectal sepsis. Br. J. Surg..

[27] Schwartz D.A., Loftus E.V., Tremaine W.J., Panaccione R., Harmsen W.S., Zinsmeister A.R., Sandborn W.J. (2002). The natural history of fistulizing Crohn’s disease in Olmsted County, Minnesota. Gastroenterology.

[28] Eglinton T.W., Barclay M.L., Gearry R.B., Frizelle F.A. (2012). The spectrum of perianal Crohn’s disease in a population-based cohort. Dis. Colon Rectum.

[29] Hellers G., Bergstrand O., Ewerth S., Holmstrom B. (1980). Occurrence and outcome after primary treatment of anal fistulae in Crohn’s disease. Gut.

[30] Mak W.Y., Mak O.S., Lee C.K., Tang W., Leung W.K., Wong M.T.L., Sze A.S.F., Li M., Leung C.M., Lo F.H. (2018). Significant Medical and Surgical Morbidity in Perianal Crohn’s Disease: Results from a Territory-Wide Study. J. Crohns Colitis.

[31] Park S.H., Aniwan S., Scott Harmsen W., Tremaine W.J., Lightner A.L., Faubion W.A., Loftus E.V. (2019). Update on the Natural Course of Fistulizing Perianal Crohn’s Disease in a Population-Based Cohort. Inflamm. Bowel Dis..

[32] Zeitz J., Fournier N., Labenz C., Biedermann L., Frei P., Misselwitz B., Scharl S., Vavricka S.R., Sulz M.C., Fried M. (2017). Risk Factors for the Development of Fistulae and Stenoses in Crohn Disease Patients in the Swiss Inflammatory Bowel Disease Cohort. Inflamm. Intest Dis..

[33] Chun J., Im J.P., Kim J.W., Lee K.L., Choi C.H., Kim H., Cheon J.H., Ye B.D., Kim Y.H., Kim Y.S. (2018). Association of Perianal Fistulas with Clinical Features and Prognosis of Crohn’s Disease in Korea: Results from the CONNECT Study. Gut Liver.

[34] Sainio P. (1984). Fistula-in-ano in a defined population. Incidence and epidemiological aspects. Ann. Chir. Gynaecol..

[35] Eisenhammer S. (1985). Emergency fistulectomy of the acute primary anorectal cryptoglandular intermuscular abscess-fistula in ano. S. Afr. J. Surg..

[36] Emile S.H., Elfeki H., Abdelnaby M. (2016). A systematic review of the management of anal fistula in infants. Tech. Coloproctol..

[37] Hamadani A., Haigh P.I., Liu I.L., Abbas M.A. (2009). Who is at risk for developing chronic anal fistula or recurrent anal sepsis after initial perianal abscess?. Dis. Colon Rectum.

[38] Seow-Choen F., Ho J.M. (1994). Histoanatomy of anal glands. Dis. Colon Rectum.

[39] Goligher J.C., Ellis M., Pissidis A.G. (1967). A critique of anal glandular infection in the aetiology and treatment of idiopathic anorectal abscesses and fistulas. Br. J. Surg..

[40] Mitalas L.E., van Onkelen R.S., Monkhorst K., Zimmerman D.D., Gosselink M.P., Schouten W.R. (2012). Identification of epithelialization in high transsphincteric fistulas. Tech. Coloproctol..

[41] American Gastroenterological Association (2003). American Gastroenterological Association medical position statement: Perianal Crohn’s disease. Gastroenterology.

[42] Kelley K.A., Kaur T., Tsikitis V.L. (2017). Perianal Crohn’s disease: Challenges and solutions. Clin. Exp. Gastroenterol..

[43] McColl I. (1967). The comparative anatomy and pathology of anal glands. Arris and Gale lecture delivered at the Royal College of Surgeons of England on 25th February 1965. Ann. R Coll. Surg. Engl..

[44] Sordo-Mejia R., Gaertner W.B. (2014). Multidisciplinary and evidence-based management of fistulizing perianal Crohn’s disease. World J. Gastrointest. Pathophysiol..

[45] Zhang M., Sun K., Wu Y., Yang Y., Tso P., Wu Z. (2017). Interactions between Intestinal Microbiota and Host Immune Response in Inflammatory Bowel Disease. Front Immunol..

[46] Petagna L., Antonelli A., Ganini C., Bellato V., Campanelli M., Divizia A., Efrati C., Franceschilli M., Guida A.M., Ingallinella S. (2020). Pathophysiology of Crohn’s disease inflammation and recurrence. Biol. Direct..

[47] Bruckner R.S., Spalinger M.R., Barnhoorn M.C., Feakins R., Fuerst A., Jehle E.C., Rickenbacher A., Turina M., Niechcial A., Lang S. (2021). Contribution of CD3+CD8- and CD3+CD8+ T Cells to TNF-alpha Overexpression in Crohn Disease-Associated Perianal Fistulas and Induction of Epithelial-Mesenchymal Transition in HT-29 Cells. Inflamm. Bowel Dis..

[48] Maggi L., Capone M., Giudici F., Santarlasci V., Querci V., Liotta F., Ficari F., Maggi E., Tonelli F., Annunziato F. (2013). CD4+CD161+ T lymphocytes infiltrate Crohn’s disease-associated perianal fistulas and are reduced by anti-TNF-alpha local therapy. Int. Arch. Allergy Immunol.

[49] van Unen V., Li N., Molendijk I., Temurhan M., Hollt T., van der Meulen-de Jong A.E., Verspaget H.W., Mearin M.L., Mulder C.J., van Bergen J. (2016). Mass Cytometry of the Human Mucosal Immune System Identifies Tissue- and Disease-Associated Immune Subsets. Immunity.

[50] Becker M., de Krijger M., Bemelman W., de Jonge W., Buskens C., Wildenberg M., Dige S.T. (2021). DOP24 Crohn’s Disease fistula show skewed lymphoid/myeloid balance, altered myeloid cell profiles and high TNF-α expression. J. Crohn’s Colitis.

[51] van Onkelen R.S., Gosselink M.P., van Meurs M., Melief M.J., Schouten W.R., Laman J.D. (2016). Pro-inflammatory cytokines in cryptoglandular anal fistulas. Tech. Coloproctol..

[52] Ratto C., Litta F., Lucchetti D., Parello A., Boninsegna A., Arena V., Donisi L., Calapa F., Sgambato A. (2016). Immunopathological characterization of cryptoglandular anal fistula: A pilot study investigating its pathogenesis. Color. Dis. Off. J. Assoc. Coloproctol. Great Br. Irel..

[53] Barnhoorn M., Schepers K., Verspaget H., Fibbe W., Hawinkels L., van Pel M., van der Meulen-de Jong A. (2019). P040 The cytokine milieu in patients with inflammatory bowel disease impacts the phenotype of mesenchymal stromal cells. J. Crohn’s Colitis.

[54] Chen T., You Y., Jiang H., Wang Z.Z. (2017). Epithelial-mesenchymal transition (EMT): A biological process in the development, stem cell differentiation, and tumorigenesis. J. Cell Physiol..

[55] Bataille F., Rohrmeier C., Bates R., Weber A., Rieder F., Brenmoehl J., Strauch U., Farkas S., Furst A., Hofstadter F. (2008). Evidence for a role of epithelial mesenchymal transition during pathogenesis of fistulae in Crohn’s disease. Inflamm. Bowel Dis..

[56] Scharl M., Weber A., Furst A., Farkas S., Jehle E., Pesch T., Kellermeier S., Fried M., Rogler G. (2011). Potential role for SNAIL family transcription factors in the etiology of Crohn’s disease-associated fistulae. Inflamm. Bowel Dis..

[57] Scharl M., Frei S., Pesch T., Kellermeier S., Arikkat J., Frei P., Fried M., Weber A., Jehle E., Ruhl A. (2013). Interleukin-13 and transforming growth factor beta synergise in the pathogenesis of human intestinal fistulae. Gut.

[58] Frei S.M., Pesch T., Lang S., Weber A., Jehle E., Vavricka S.R., Fried M., Rogler G., Scharl M. (2013). A role for tumor necrosis factor and bacterial antigens in the pathogenesis of Crohn’s disease-associated fistulae. Inflamm. Bowel Dis..

[59] Kirkegaard T., Hansen A., Bruun E., Brynskov J. (2004). Expression and localisation of matrix metalloproteinases and their natural inhibitors in fistulae of patients with Crohn’s disease. Gut.

[60] Glassner K.L., Abraham B.P., Quigley E.M.M. (2020). The microbiome and inflammatory bowel disease. J. Allergy Clin. Immunol..

[61] Haac B.E., Palmateer N.C., Seaton M.E., Van Y.R., Fraser C.M., Bafford A.C. (2019). A Distinct Gut Microbiota Exists Within Crohn’s Disease-Related Perianal Fistulae. J. Surg. Res..

[62] Tozer P.J., Rayment N., Hart A.L., Daulatzai N., Murugananthan A.U., Whelan K., Phillips R.K. (2015). What role do bacteria play in persisting fistula formation in idiopathic and Crohn’s anal fistula?. Color. Dis. Off. J. Assoc. Coloproctol. Great Br. Irel..

[63] Radlmayr M., Torok H.P., Martin K., Folwaczny C. (2002). The c-insertion mutation of the NOD2 gene is associated with fistulizing and fibrostenotic phenotypes in Crohn’s disease. Gastroenterology.

[64] Zhu J., Wang Q., Mei Z. (2021). Preliminary study on the pathogenesis of anal fistula. medRxiv.

[65] Eykyn S.J., Grace R.H. (1986). The relevance of microbiology in the management of anorectal sepsis. Ann. R Coll Surg. Engl.

[66] Toyonaga T., Matsushima M., Tanaka Y., Shimojima Y., Matsumura N., Kannyama H., Nozawa M., Hatakeyama T., Suzuki K., Yanagita K. (2007). Microbiological analysis and endoanal ultrasonography for diagnosis of anal fistula in acute anorectal sepsis. Int. J. Color. Dis..

[67] Leung E., McArdle K., Yazbek-Hanna M. (2009). Pus swabs in incision and drainage of perianal abscesses: What is the point?. World J. Surg..

[68] Xu R.W., Tan K.K., Chong C.S. (2016). Bacteriological study in perianal abscess is not useful and not cost-effective. ANZ J. Surg..

[69] van Onkelen R.S., Mitalas L.E., Gosselink M.P., van Belkum A., Laman J.D., Schouten W.R. (2013). Assessment of microbiota and peptidoglycan in perianal fistulas. Diagn. Microbiol. Infect. Dis..

[70] Spinelli A., Armuzzi A., Ciccocioppo R., Danese S., Gionchetti P., Luglio G., Orlando A., Rispo A., Rizzello F., Sofo L. (2020). Management of patients with complex perianal fistulas in Crohn’s disease: Optimal patient flow in the Italian clinical reality. Dig. Liver Dis..

[71] Yarur A.J., Kanagala V., Stein D.J., Czul F., Quintero M.A., Agrawal D., Patel A., Best K., Fox C., Idstein K. (2017). Higher infliximab trough levels are associated with perianal fistula healing in patients with Crohn’s disease. Aliment Pharm..

[72] Adegbola S.O., Sarafian M., Sahnan K., Pechlivanis A., Phillips R.K.S., Warusavitarne J., Faiz O., Haddow J., Knowles C., Tozer P. (2022). Lack of anti-TNF drugs levels in fistula tissue—a reason for nonresponse in Crohn’s perianal fistulating disease?. Eur. J. Gastroenterol. Hepatol..

[73] Chudy-Onwugaje K.O., Christian K.E., Farraye F.A., Cross R.K. (2019). A State-of-the-Art Review of New and Emerging Therapies for the Treatment of IBD. Inflamm. Bowel Dis..

[74] Feagan B.G., Schwartz D., Danese S., Rubin D.T., Lissoos T.W., Xu J., Lasch K. (2018). Efficacy of Vedolizumab in Fistulising Crohn’s Disease: Exploratory Analyses of Data from GEMINI 2. J. Crohn’s Colitis.

[75] Attauabi M., Burisch J., Seidelin J.B. (2021). Efficacy of Ustekinumab for Active Perianal Fistulizing Crohn Disease: A Double-Center Cohort Study. Inflamm. Bowel Dis..

[76] de Groof E.J., Cabral V.N., Buskens C.J., Morton D.G., Hahnloser D., Bemelman W.A., the Research Committee of the European Society of Coloproctology (2016). Systematic review of evidence and consensus on perianal fistula: An analysis of national and international guidelines. Color. Dis. Off. J. Assoc. Coloproctol. Great Br. Irel..

[77] Ommer A., Herold A., Berg E., Furst A., Sailer M., Schiedeck T. (2011). Cryptoglandular anal fistulas. Dtsch Arztebl Int..

[78] van der Hagen S.J., Baeten C.G., Soeters P.B., Beets-Tan R.G., Russel M.G., van Gemert W.G. (2005). Staged mucosal advancement flap for the treatment of complex anal fistulas: Pretreatment with noncutting Setons and in case of recurrent multiple abscesses a diverting stoma. Color. Dis..

[79] Stellingwerf M.E., van Praag E.M., Tozer P.J., Bemelman W.A., Buskens C.J. (2019). Systematic review and meta-analysis of endorectal advancement flap and ligation of the intersphincteric fistula tract for cryptoglandular and Crohn’s high perianal fistulas. BJS Open.

[80] Rojanasakul A. (2009). LIFT procedure: A simplified technique for fistula-in-ano. Tech. Coloproctol..

[81] Yassin N.A., Hammond T.M., Lunniss P.J., Phillips R.K. (2013). Ligation of the intersphincteric fistula tract in the management of anal fistula. A systematic review. Color. Dis..

[82] Schwandner O. (2013). Video-assisted anal fistula treatment (VAAFT) combined with advancement flap repair in Crohn’s disease. Tech. Coloproctol..

[83] Wilhelm A., Fiebig A., Krawczak M. (2017). Five years of experience with the FiLaC laser for fistula-in-ano management: Long-term follow-up from a single institution. Tech. Coloproctol..

[84] Elfeki H., Shalaby M., Emile S.H., Sakr A., Mikael M., Lundby L. (2020). A systematic review and meta-analysis of the safety and efficacy of fistula laser closure. Tech. Coloproctol..

[85] Wewer M.D., Zhao M., Nordholm-Carstensen A., Weimers P., Seidelin J.B., Burisch J. (2021). The Incidence and Disease Course of Perianal Crohn’s Disease: A Danish Nationwide Cohort Study, 1997-2015. J. Crohns Colitis.

[86] Song E.M., Lee H.S., Kim Y.J., Oh E.H., Ham N.S., Kim J., Hwang S.W., Park S.H., Yang D.H., Ye B.D. (2020). Incidence and Outcomes of Perianal Disease in an Asian Population with Crohn’s Disease: A Nationwide Population-Based Study. Dig. Dis. Sci..

[87] Lansdorp C.A., Buskens C.J., Gecse K.B., Lowenberg M., Stoker J., Bemelman W.A., D’Haens G., van Hulst R.A. (2022). Hyperbaric oxygen therapy for the treatment of perianal fistulas in 20 patients with Crohn’s disease: Results of the HOT-TOPIC trial after 1-year follow-up. United Eur. Gastroenterol. J..

[88] Meima-van Praag E.M., van Rijn K.L., Wasmann K., Snijder H.J., Stoker J., D’Haens G.R., Gecse K.B., Gerhards M.F., Jansen J.M., Dijkgraaf M.G.W. (2022). Short-term anti-TNF therapy with surgical closure versus anti-TNF therapy in the treatment of perianal fistulas in Crohn’s disease (PISA-II): A patient preference randomised trial. Lancet Gastroenterol. Hepatol..

[89] Barnhoorn M.C., Wasser M., Roelofs H., Maljaars P.W.J., Molendijk I., Bonsing B.A., Oosten L.E.M., Dijkstra G., van der Woude C.J., Roelen D.L. (2020). Long-term evaluation of allogeneic bone marrow-derived mesenchymal stromal cell therapy for Crohn’s disease perianal fistulas. J. Crohn’s Colitis.

[90] Panes J., Garcia-Olmo D., Van Assche G., Colombel J.F., Reinisch W., Baumgart D.C., Dignass A., Nachury M., Ferrante M., Kazemi-Shirazi L. (2016). Expanded allogeneic adipose-derived mesenchymal stem cells (Cx601) for complex perianal fistulas in Crohn’s disease: A phase 3 randomised, double-blind controlled trial. Lancet.

[91] Panes J., Garcia-Olmo D., Van Assche G., Colombel J.F., Reinisch W., Baumgart D.C., Dignass A., Nachury M., Ferrante M., Kazemi-Shirazi L. (2018). Long-term Efficacy and Safety of Stem Cell Therapy (Cx601) for Complex Perianal Fistulas in Patients With Crohn’s Disease. Gastroenterology.

[92] Nagaishi K., Arimura Y., Fujimiya M. (2015). Stem cell therapy for inflammatory bowel disease. J. Gastroenterol..

[93] Molendijk I., Bonsing B.A., Roelofs H., Peeters K.C., Wasser M.N., Dijkstra G., van der Woude C.J., Duijvestein M., Veenendaal R.A., Zwaginga J.J. (2015). Allogeneic Bone Marrow-Derived Mesenchymal Stromal Cells Promote Healing of Refractory Perianal Fistulas in Patients With Crohn’s Disease. Gastroenterology.

[94] Hirsch R.D., Keung C., Con D., Vasudevan A., Van Langenberg D.R., Niewiadomski O. (2022). Direct health care costs of managing perianal Crohn’s Disease in a population based cohort. Scand J. Gastroenterol..

[95] Wilson J.C., Furlano R.I., Jick S.S., Meier C.R. (2016). Inflammatory Bowel Disease and the Risk of Autoimmune Diseases. J. Crohn’s Colitis.

[96] Forabosco P., Bouzigon E., Ng M.Y., Hermanowski J., Fisher S.A., Criswell L.A., Lewis C.M. (2009). Meta-analysis of genome-wide linkage studies across autoimmune diseases. Eur. J. Hum Genet.

[97] Moller F.T., Andersen V., Wohlfahrt J., Jess T. (2015). Familial risk of inflammatory bowel disease: A population-based cohort study 1977-2011. Am J. Gastroenterol..

[98] Chao C.Y., Bessissow T. (2018). Does Familial IBD Have its Own Signature?. J. Crohns Colitis.

[99] Gecse K., Khanna R., Stoker J., Jenkins J.T., Gabe S., Hahnloser D., D’Haens G. (2013). Fistulizing Crohn’s disease: Diagnosis and management. United Eur. Gastroenterol. J..

[100] Buchanan G.N., Halligan S., Bartram C.I., Williams A.B., Tarroni D., Cohen C.R. (2004). Clinical examination, endosonography, and MR imaging in preoperative assessment of fistula in ano: Comparison with outcome-based reference standard. Radiology.

[101] Stevens T.W., D’Haens G.R., Duijvestein M., Bemelman W.A., Buskens C.J., Gecse K.B. (2019). Diagnostic accuracy of faecal calprotectin in patients with active perianal fistulas. United Eur. Gastroenterol. J..

[102] Bakan S., Olgun D.C., Kandemirli S.G., Tutar O., Samanci C., Dikici S., Simsek O., Rafiee B., Adaletli I., Mihmanli I. (2015). Perianal Fistula With and Without Abscess: Assessment of Fistula Activity Using Diffusion-Weighted Magnetic Resonance Imaging. Iran J. Radiol.

